# The Effect of Family Voice Interventions on Delirium Incidence and Duration in Adult ICU Patients: A Systematic Review and Meta‐Analysis

**DOI:** 10.1111/nicc.70492

**Published:** 2026-04-14

**Authors:** Ella Peschel, Marion Diegelmann, Roland Eßl‐Maurer, Pia Goetze, Susanne Krotsetis, Anemone Neumann‐Wagner, Eunkyeong Oh, Bianca Schmidt‐Maciejewski, Florian Schimböck, Vanessa Vater, Peter Nydahl

**Affiliations:** ^1^ University Hospital of Schleswig‐Holstein Lübeck Germany; ^2^ Nursing Research University Hospital Frankfurt Germany; ^3^ Nursing Department, Coordination Development Clinical Nursing Practice, University Hospital Salzburg Paracelsus Medical University Salzburg Austria; ^4^ University Hospital of Schleswig‐Holstein Kiel Germany; ^5^ Department of Psychiatry and Psychotherapy University Medical Center Hamburg Eppendorf (UKE) Hamburg Germany; ^6^ Asklepios Hospital St. Georg, Department of Nursing Competence and Development Hamburg Germany; ^7^ Institute of General Practice University Hospital of Schleswig‐Holstein/University Kiel Germany; ^8^ Nursing Research and Development University Hospital of Schleswig‐Holstein Kiel Germany; ^9^ Nursing Science and Development Paracelsus Medical University Salzburg Austria

**Keywords:** critical care, delirium, family, family involvement, patient‐centred care, prevention

## Abstract

**Background:**

Delirium is a common and serious complication in patients on intensive care units (ICUs). Family voice interventions may support orientation, reduce sensory deprivation and mitigate delirium, but their efficacy remains unclear.

**Aim:**

The primary aim was to systematically evaluate the effects of family voice interventions on the incidence and duration of delirium in adult ICU patients.

**Methods:**

We searched PubMed, CINAHL, Embase, Cochrane Library and APA PsycNet and grey literature from inception to present. Eligible randomized controlled trials (RCT) included adults (≥ 18 years) in ICU settings receiving family voice interventions via live or recorded voices of family members. Primary outcomes were delirium incidence and duration, assessed using validated tools. Quality assessment was conducted by using RoB 2.0 and Grade. Meta‐analyses were performed using RevMan 9.5.1.

**Results:**

Out of 438 titles, seven RCT with a low to high risk of bias including 582 patients were analysed. Certainty of evidence was moderate. Meta‐analysis evaluating family voice interventions versus usual care showed a significant reduction in delirium incidence (OR: 0.39 [CI: 0.26, 0.58], *p* < 0.001; *χ* ^2^ = 3.09, *I* ^2^ = 0%, *p* = 0.54) and duration (MD −0.90 days CI [−1.28, −0.53], *χ* ^2^ = 0.69, *I* ^2^ = 0%; *p* = 0.71).

**Conclusions:**

Family voice interventions may aid in preventing and reducing delirium in critically ill patients, potentially supporting patient recovery and improving family psychological well‐being. Given the moderate certainty of evidence, more robust research is necessary.

**Relevance for Clinical Practice:**

Critically ill patients and families should be educated about delirium. Clinicians should integrate families in the prevention and treatment of delirium by using live or recorded re‐orienting voice interventions.

## Impact Statements

- What is known about this topic
  ○ Family voice interventions help reorient patients to persons, time and place.
  ○ Familiar language and tone facilitate understanding and provide insight to patients' needs and fears.
  ○ Empathy, personal topics and familiar sounds create a sense of safety and strengthen mental well‐being.
- What this paper adds
  ○ Family members should communicate regularly with the patient to support reorientation, provide a sense of safety and help establish a day–night rhythm.
  ○ Family voice interventions can be used in critically ill patients for prevention and treatment of delirium.
  ○ The effects of family voice interventions on delirium severity and burden, hallucinations, long‐term psychological impairments, safety, length of stay in ICU and hospital, duration of mechanical ventilation and quality of life require further research.

## 1 Introduction

Delirium is a common and serious neuropsychiatric condition in hospitalized patients, particularly those in intensive care units (ICUs) [1]. It is characterized by an acute and fluctuating disturbance in attention, awareness and cognition, often accompanied by psychomotor changes, sleep–wake cycle disruption and affective symptoms [2]. Occurring at a prevalence of 50%–80% in mechanically ventilated ICU patients [3, 4]. Delirium arises from a complex interplay of predisposing vulnerabilities—such as advanced age, frailty, cognitive impairment and acute precipitating factors including sepsis, surgery, hypoxia, medication and effects [5]. Its pathophysiology includes neuroinflammatory responses, impaired neurotransmitter regulation, brain energy deficits and disrupted neuronal network connectivity [1, 6].

The occurrence of delirium is strongly associated with adverse outcomes, including prolonged ICU and hospital length of stay, increased risk of long‐term cognitive impairment, institutionalization and higher mortality [7, 8, 9]. Moreover, persistent and subsyndromal forms of delirium are increasingly recognized and carry prognostic significance [10]. Although pharmacological interventions such as antipsychotics have been widely used, their effectiveness in preventing or treating ICU delirium is limited [11, 12]. Consequently, current guidelines and research recommend multicomponent, non‐pharmacological strategies as the most effective approach, including minimizing sedation, promoting mobility, ensuring orientation and involving family members in patient care [13, 14, 15, 16, 17].

Recent research has highlighted the importance of humanizing intensive care and re‐establishing meaningful connections between patients and their loved ones [18, 19, 20, 21]. Families are defined by patients as individuals with whom patients have a significant relationship [22]. Families may enhance orientation, reduce sensory deprivation, and provide emotional reassurance, potentially influencing the course of delirium [23]. Family members' voices have emerged as a promising intervention for preventing ICU patients from delirium and in reducing delirium duration [24, 25, 26].

Several systematic reviews have examined family involvement or family presence in critical care, reporting potential benefits for patient outcomes but also considerable heterogeneity in study populations, interventions and outcome measures. Many of these reviews address broad concepts of family‐centred care or include diverse non‐pharmacological interventions, making it difficult to draw conclusions about specific mechanisms or intervention components [27, 28]. Specifically, a systematic review focussed on family voice interventions found reductions in delirium incidence and duration across multiple randomized controlled trials but did not include the assessment of certainty of evidence [26]. Additional RCTs have since been published, and previous reviews differ in inclusion criteria and intervention characteristics, limiting comparability and clinical applicability [29, 30]. Consequently, a focussed and updated synthesis of randomized controlled trials on family voice interventions and assessment of certainty of evidence is needed. The present review aims to address this gap by providing an updated, methodologically rigorous evaluation of the impact of family voice interventions in the incidence and duration in adult ICU patients.

## 2 Methods

This systematic review was conducted in accordance with the PRISMA guidelines for reporting and followed the methodological standards of the Cochrane Handbook for Systematic Reviews of Interventions to ensure rigour throughout the process [31, 32] (Table S2). The protocol has been developed in advance and registered in PROSPERO (CRD420251046497).

### 2.1 Eligibility Criteria

Studies were included based on the following pre‐defined criteria, structured according to the PICOS framework:

- Population: adult patients (≥ 18 years) admitted to an intensive care unit (ICU).
- Intervention: auditory stimulation using the voice recordings or live voices of family members (e.g., parents, children, spouses) as single intervention or as part of bundles.
- Comparator: Standard ICU care or usual care without family voice intervention, auditory stimuli or placebo.
- Outcomes: the primary outcomes were the incidence and duration of delirium, which were assessed using validated tools (e.g., CAM‐ICU, ICDSC, DSM criteria). Secondary outcomes included delirium severity, duration of sedation, ICU length of stay, mechanical ventilation, stay in hospital and safety, which was defined as any unwanted event causing harm to patients.
- Study design: randomized controlled trials (RCTs) were included. Systematic reviews (e.g., including bundles for delirium prevention and therapy) were used for reference screening.
- Languages and publication date: no restrictions for languages and publication date.

Excluded were quasi‐experimental studies, stepped‐wedge RCTs, quality improvement projects, case reports involving ≤ 10 patients, editorials, narrative reviews and others. Only randomized controlled trials (RCTs) were included, as they provide the most reliable evidence for intervention effects.

### 2.2 Information Sources and Search Strategy

The following electronic databases were systematically searched: PubMed (MEDLINE), CINAHL (EBSCOhost), Cochrane Central Register of Controlled Trials (CENTRAL), APA PsycNet, Embase, grey literature, Google Scholar and backward screening of reference lists of included studies. The search strategy included a combination of controlled vocabulary (e.g., MeSH terms) and free‐text keywords related to delirium (e.g., ‘delirium’, ‘confusion’), intensive care (e.g., ‘critical care’, ‘intensive care unit’) and family (e.g., family’, ‘relatives’) and voice (e.g., ‘voices’, ‘auditory stimulation’) (S1). The search strategies were adapted to the specific requirements of each database and were then peer‐reviewed by a librarian using the PRESS checklist [33]. No restrictions were applied regarding the publication date or language. The search was performed between 08 May 2025 and 19 May 2025 with an update on 20 November 2025.

### 2.3 Study Selection and Data Extraction

All records were imported into reference management software (Rayyan), followed by the removal of duplicates. Two independent reviewers (EO and FS) screened titles and abstracts for eligibility. Full texts of potentially relevant studies were screened for inclusion. Any discrepancies were resolved through discussion with a third reviewer (PN).

A pre‐defined standardized data extraction form was used to collect relevant study characteristics, including details of the population, intervention components, outcome measures and results. Two reviewers (MD and BSM) extracted these characteristics independently. In studies with three groups, the results of both intervention groups were combined. In studies with three groups, the results were pooled to avoid double counting in the control group by adding comparable interventions into one intervention group.

### 2.4 Risk of Bias Assessment

The risk of bias was assessed independently by two reviewers (VV and REM) using the Cochrane Risk of Bias tool for randomized controlled trials (RoB 1). Each study was evaluated at the study level across the following domains: the randomization process, deviations from intended interventions, missing outcome data, measurement of the outcome and selective reporting. These domains correspond to the key sources of bias assessed in the Cochrane RoB 1 tool. An overall risk of bias judgement (low, unclear or high) was assigned to each study [34, 35]. Any disagreements were resolved through discussion or by consultation with a third reviewer (PN).

### 2.5 Certainty of Evidence (GRADE)

The certainty of evidence for each outcome was assessed using the Grading of Recommendations Assessment, Development and Evaluation (GRADE) approach [36]. For the GRADE assessment, risk of bias was considered at the study level, as outcome‐specific risk of bias assessments were not performed. Two reviewers independently evaluated the body of evidence across five domains: risk of bias, inconsistency, indirectness, imprecision and publication bias. The certainty of evidence was rated as high, moderate, low or very low [37]. Any disagreements were resolved through discussion.

### 2.6 Data Extraction and Statistical Analysis

All quantitative analyses were conducted using Review Manager (RevMan) version 9.5.1. The analysis was followed by the guidelines for conducting meta‐analyses of both dichotomous and continuous outcomes, in line with the Cochrane Handbook for Systematic Reviews of Interventions [38].

For dichotomous outcomes (e.g., incidence of delirium), the pooled risk ratios (RRs) with 95% confidence intervals (CIs) were calculated. In addition, for continuous outcomes (e.g., duration of delirium in days), pooled mean differences (MDs) or standardized mean differences (SMDs) (if different measurement scales were used) were computed with 95% CIs. To enable inclusion in the meta‐analysis, if median and interquartile range were reported, they were converted into mean and standard deviation using the established method by Wan et al. [39, 40, 41].

A random effects model was used to account for potential heterogeneity between studies caused by clinical and methodological differences. Statistical heterogeneity was evaluated using the *I* ^2^ statistic, with values over ≥ 50% representing considerable heterogeneity [42].

Further sub‐analyses for heterogeneity, publication bias, or interesting variables such as live voice vs. recorded voice were planned but could not be performed due to the limited number of included studies.

## 3 Results

**FIGURE 1:**
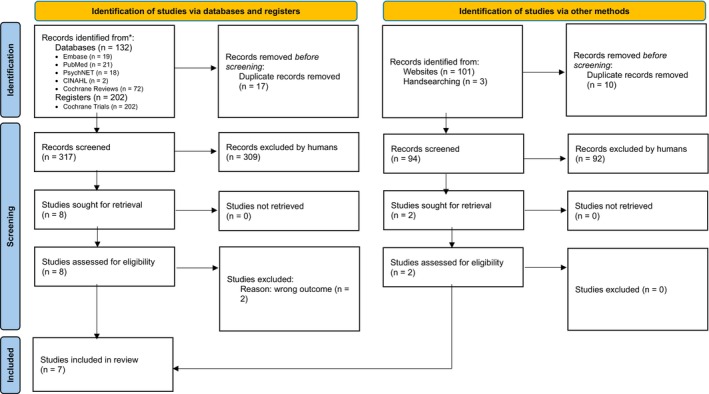
Preferred Reproting Item for Systematic Reviews and Meta‐Analyses (PRISMA) Flowchart. Source: M. J. Page, et al., *BMJ* 372 (2021): n71, https://doi.org/10.1136/bmj.n71 [32]. This work is licensed under CC BY 4.0. To view a copy of this licence, visit https://creativecommons.org/licenses/by/4.0/.

The systematic literature review identified 334 references across six data sources. An additional 104 references were revealed through alternative search strategies. In total, seven articles were included in the analysis after duplicates were removed and screening was conducted based on eligibility criteria (Figure 1). Some promising studies had to be excluded, due to not fulfilling the inclusion criteria [43, 44, 45].

### 3.1 Characteristics of Included Studies

The seven RCT were published between 2017 and 2025. Three of the studies were performed in China [46, 47, 48], two in the USA [24, 29], one in Turkey [49] and one in Germany [30]. In total, the studies included 582 patients with an average age ranging from 55 to 61 years.

The standard of care was used as the comparison in all studies. Three studies used a three‐group comparison [24, 46, 49]; such as structured vs. unstructured family voices [46], structured family voices vs. structured statements of unknown voices [24], and structured family voices with random voices. All studies also used standard of care as a third group, while the other studies only compared family voices with the standard of care [29, 30, 47, 48]. Three studies used family messages as a single intervention, while one study described the intervention as part of a bundle [47] (Table 1).

**TABLE 1:** Description of the included studies.

Study, country^a^	Patient total *n* (%)	Patient age in years^b^	Patient female *n* (%)	Def of family	Voice	Comparator	Duration (freq, minutes, days)
Kasapoğlu & Enҫ, Turkey	94	Not reported	43 (40.2%)	Voice of a family member (not precisely defined)	g.1: other voice g.2: Voice recorded (orientation message) Voice present (newspaper)	Usual care	Three consecutive days, two times a day at 10 am and 4 pm (orientation information) for 5–10 min newspaper reading after orientation message in the morning
Lin et al., China	80	61.04 (±7.59)	32 (40%)	Family caregivers older than 18 years, who took primary responsibility for caring	Present Voice at ICU, psychological and orientation messages; cognitive Stimulation; ICU Care Participation	Usual care	30 min twice a day; 8:00 am and 16:00 pm psych. Support: 6–8 min. Orientation: 4–6 min. Cognitive simulation: 4–6 min. Participation in care: 8–15 min. Visits: 4.15 (SD = 1.31) exp. group 4.55 (SD = 2.06) contr. group
Ma et al., China	213	g.A: 55.0 (±17.5) gB: 59.9 (+18.2) g.C: 56.9 (+16.4)	152 (28.6%)	Adults (older than or equal to 18 years); lived with the patient; primary caregiver among the patient's family	g.A: structured family voice stimulation: Introduction speaker, orient messages, Explanation environment, emotional support g.B: unstructured family voice stimulation (content freely chosen)	Usual care (no family visits: COVID‐19)	5 days 5 min Once every 2 h, from 8 am to 16 pm
Munro et al., USA	30	59.5 (±17)	11 (36.7%)	A family member of family's choice	g.1: unknown voices, same content g.2: structured family voice content	Usual care	3 days Max. 2 min long Every hour for 8 h/day, between 9 am and 16 pm
Sprügel et al., Germany	45	60 (53–68)	20 (44%)	Individuals with a significant relationship according the SCCM Guideline	Recorded structured family voices, regarding the patient's condition and the request to breathe in and out	Neutral, non‐human sounds (ambient ICU noises)	10 min of audio/day, Segment was played three times daily (6–8 am, 12–14 pm, 18–20 pm) during assisted mechanical ventilation; intervention till patient was extubated/weaned
Munro et al., USA	178	59.3 (17.1)	71 (40%)	A family member of choice	Structured recorded family voices, including general information about environment, visual and auditory stimuli	Usual care	2‐min messages played hourly during usual daytime hours (9 AM–4 PM) max. 5 days or until ICU discharge
Liang et al., China	152	56.2 (± 16.4)	60 (39.5%)	A family member of choice	Family members recorded reorientation messages	Usual care	7 days (one 45‐min session daily: 15 min pre‐bedside preparation, 30 min bedside sensory stimulation)

Abbreviations: g, group; SCCM, Society of Critical Care Medicine.

^a^ All studies were randomized controlled trials using the confusion assessment method for the Intensive Care Unit as assessment for delirium.

^b^ Reported as mean (standard deviation) or median (interquartile range).

The duration of family involvement varied, ranging from three consecutive days twice a day at 10 a.m. and 4 p.m. for 30 min, to 5 min at two‐hourly intervals for 5 days, to a maximum of 2 min every hour for 8 h over 3 days or until ICU discharge (Table S3).

Four studies reported delirium incidence [24, 30, 47, 49], two studies reported the length of delirium [46, 48] and one reported delirium free days [29]. The presence of delirium was measured in all studies using the confusion Assessment Method for the Intensive Care Unit (CAM‐ICU) (Table 1).

### 3.2 Secondary Outcome Parameters

Secondary parameters such as ICU length of stay, duration of mechanical ventilation, and delirium severity were reported in few studies. Parameters such as days in hospital, unwanted safety events, duration of sedation and mortality were not reported (Table 2).

**TABLE 2:** Description of the secondary outcome parameter of the included studies.

Study^a^	Delirium severity	Days on MV	Days in ICU	Days in hospital	Unwanted safety events	Duration of sedation
Kasapoğlu & Enҫ, 2022	n.r.	n.r.	n.r.	n.r.	n.r.	n.r.
Lin et al., 2023	n.r.	I: 0.82 (0.59–1.49) vs. C: 0.85 (0.68–1.59)	I: 1.91 (1.68–2.81) vs. C: 2.77 (2–3.64)	n.r.	n.r.	n.r.
Ma et al.^b^, 2025	I1: 3.1 (0.4) vs. I2: 4.0 (0.3) vs. C: 5.1 (0.3)	n.r.	n.r.	n.r.	n.r.	n.r.
Munro et al.^b^, 2017	n.r.	I1: 0.8 (0.8) vs. I2: 4.1 (9.0) vs. C: 1.2 (2.7)	I1: 3 (1.6) vs. I2: 7.1 (9.4) vs. C: 4.9 (3.5)	n.r.	n.r.	n.r.
Sprügel et al., 2025	n.r.	I: 0.94 (0.55–1.56) vs. C: 1.84 (1.25–2.68)	n.r.	n.r.	n.r.	n.r.
Munro et al., 2025	n.r.	n.r.	n.r.	n.r.	n.r.	n.r.
Liang et al., 2023	I: 3.70 (+1.25) vs. C: 5.68 (+1.57)	I: 0.83 (2.25) vs. C: 0.92 (1.46)	I: 3.88 (+2.61) vs. C: 3.96 (+2.85)	n.r.	n.r.	n.r.

Abbreviations: C, control group; ICU, Intensive Care Unit; I, intervention group; MV, mechanically ventilation; n.r., not reported; RCT, randomized controlled trail.

^a^ Reported as mean (standard deviation) or median (interquartile range).

^b^ RCT with two intervention groups.

The outcome safety events were reported by Lin [47] only, with no adverse events or complications at all, including unplanned extubations, nosocomial infections or abnormal vital signs.

### 3.3 Risk of Bias

The overall risk of bias across included studies ranged from high to low, with the majority of RCTs assessed as having a low risk of bias (Table 3).

**TABLE 3:** Risk of bias judgement.

Study	Randomization process	Deviations from intended interventions	Missing outcome data	Measurement of the outcome	Selection of the reported result	Overall risk of bias
Kasapoğlu & Enҫ, 2022	Unclear	High	Unclear	Unclear	Unclear	High
Lin et al., 2023	High	Unclear	Low	Low	Low	High
Ma et al., 2025	Low	Unclear	Low	Low	Low	Low
Munro et al., 2017	Unclear	Unclear	Low	Unclear	Unclear	High
Sprügel et al., 2025	Low	Low	Low	Low	Low	Low
Munro et al., 2025	Low	Low	Low	Low	Low	Low
Liang et al., 2023	Low	Low	Low	Low	unclear	Low

### 3.4 Meta‐Analysis

**FIGURE 2:**
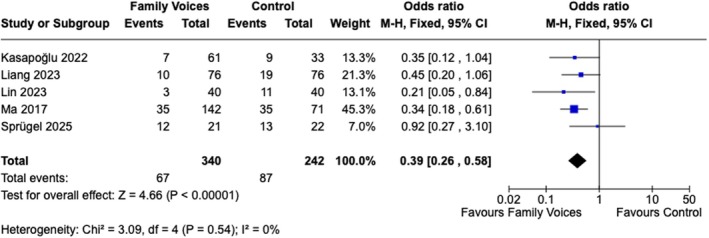
Meta‐analysis for delirium prevalence. *Family voices always includes both intervention groups if there were more than one in the study.

Overall, seven meta‐analyses were performed, involving a total of 582 patients. The meta‐analysis of the incidence of delirium included five studies with 582 patients. A fixed model was used for all meta‐analyses based on the results of the data extraction, which showed that the studies were very homogenous. This analysis showed a significant reduction in the odds ratio for delirium when reorientation messages were provided by family members (OR: 0.39 [CI: 0.26, 0.58], *p* < 0.001; χ^2^ = 3.09, *I* ^2^ = 0%, *p* = 0.54) (Figure 2). Additionally, a meta‐analysis was performed of single intervention studies only. This analysis also showed a significant reduction in the chance of delirium occurring when family voices were used as an intervention, with no significant heterogeneity (OR 0.41 [0.27, 0.63], *p* < 0.0001; χ^2^ = 2.29, *I* ^2^ = 0%, *p* = 0.51) (Figure S4).

**FIGURE 3:**
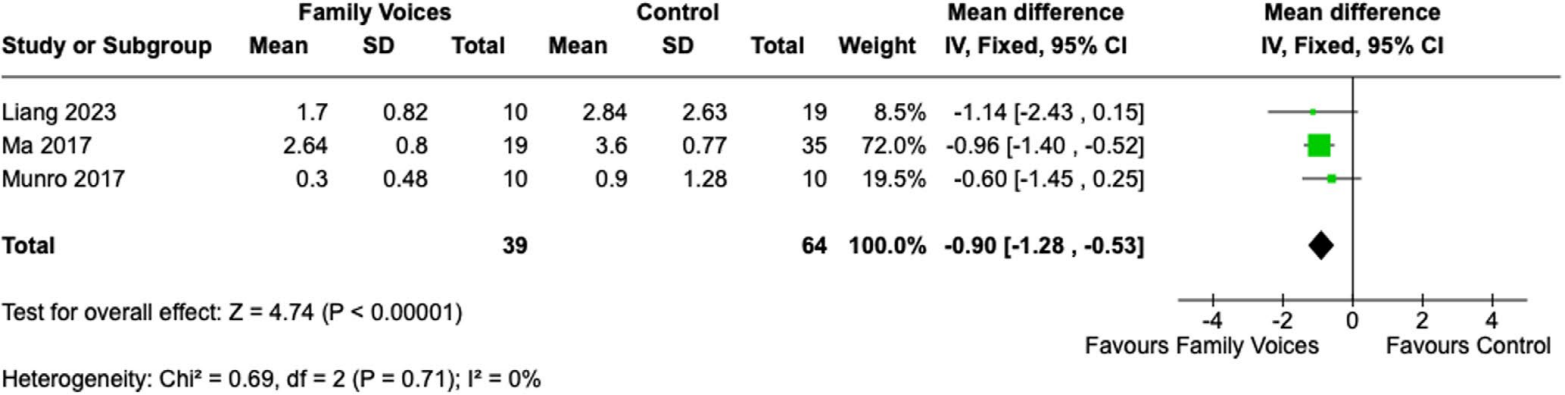
Meta‐analysis for delirium duration.

The meta‐analysis of the length of delirium included three studies with a total of 103 patients. This analysis also showed a significant reduction in the length of delirium when family voices were used as an intervention. The analysis showed mean differences of −0.90 days (CI [−1.28, −0.53], *χ* ^2^ = 0.69, *I* ^2^ = 0%; *p* = 0.71) (Figure 3, Figure S5).

In addition, four meta‐analysis were conducted for secondary outcomes. The meta‐analysis of delirium severity revealed a significant reduction of 2 points on the CAM‐ICU scale (−2.00 CI [−2.118, −1.89], *p* = 0.001; χ^2^ = 0.01, *I* ^2^ = 0%; *p* = 0.93) (Figure S6). Another analysis examining the duration of mechanical ventilation (MV) showed a significant reduction of 0.28 days (−0.28 CI [−0.52, −0.03] *p* = 0.03; χ^2^ = 6.34, *I* ^2^ = 68%; *p* = 0.04) (Figure S7). Furthermore, the intervention group demonstrated a significant reduction in ICU length of stay compared to the control group; however, this analysis indicated substantial heterogeneity (−0.41 [−0.82, −0.00] *p* = 0.05; χ^2^ = 8.39, *I* ^2^ = 76%; *p* = 0.02) (Figure S8).

Due to the limited number of studies with a low risk of bias, further sensitivity analyses were not possible. A sensitivity analysis led to no difference between the use of a random effects or a fixed effect model.

### 3.5 Certainty of Evidence

The overall certainty of evidence for the primary outcomes of the meta‐analysis was rated as moderate according to the GRADE approach. Results for the primary outcomes are presented in the summary of findings (SoF) table (Table 4). Additional outcomes and the domain‐specific risk of bias assessments for all included studies are summarized in the evidence profile table (Table 5).

**TABLE 4:** Summary of findings for primary outcomes.

Outcome	No. of participants	Relative effect (95% CI)	Absolute effect	Certainty of evidence (GRADE)
Delirium prevalence	582 (5 RCTs)	OR 0.39 (0.26, 0.58)	Fewer cases of delirium in the intervention group	+++ Moderate^a^
Delirium duration	103 (3 RCTs)	MD −0.90 (−1.28, −0.53)	0.9 days shorter on average	+++ Moderate^b^

^a^ Downgraded one level for risk of bias due to several included studies being assessed as having a high risk of bias at the study level. No downgrading for inconsistency (*I* ^2^ = 0), indirectness (interventions and populations were comparable) or imprecision (narrow confidence interval). No upgrading applied.

^b^ Downgraded one level for risk of bias, as some included studies were assessed as high risk at the study level. No downgrading for inconsistency (*I* ^2^ = 0) or indirectness. Downgraded one level for imprecision due to small total sample size. No upgrading applied.

**TABLE 5:** Evidence profile of included studies for primary outcomes.

Outcome	Risk of Bias	Inconsistency	Indirectness	Imprecision	Effect Size	Overall Judgement
Delirium prevalence	One study high risk (downgrade 1 level)	*I* ^2^ = 0 (no downgrade)	Similar intervention (no downgrade)	Narrow CI (no downgrade)	OR = 0.32 (no upgrade due other limitations)	+++ (downgraded 1 level due study quality)
Delirium prevalence only single intervention	One study some risk (downgrade 1 level)	*I* ^2^ = 0 (no downgrade)	Similar intervention (no downgrade)	Narrow CI (no downgrade)	OR = 0.34 (no upgrade due other limitations)	+++ (downgraded 1 level due study quality)
Delirium duration	One study some risk (downgrade 1 level)	*I* ^2^ = 0 (no downgrade)	Similar intervention (no downgrade)	Narrow CI (no downgrade)	MD = −0.88 (no upgrade)	+++ (downgraded 1 level due study quality)

*Note:* ++++, high evidence; +++moderate evidence; ++, low evidence; +, very low evidence.

Abbreviations: CI, confidence Interval; GRADE, Grading of Recommendations Assessment, Development and Evaluation; OR, odds ratio; MD, mean difference.

## 4 Discussion

This systematic review provides a synthesis of the current evidence regarding the impact of family voices in delirium in critically ill patients. To address this aim, seven RCTs with a low to high risk of bias with more than 500 patients, comparing family voice interventions with usual care were included. Family voice interventions vs. usual care reduced the likelihood of delirium by nearly 70%, with no observed heterogeneity. A subgroup analysis of single‐intervention studies also showed no heterogeneity and a significant effect on the absence of delirium. Additionally, the meta‐analysis with continuous data indicated a significant reduction in the duration of delirium by nearly 1 day using family reorientation messages.

The moderate certainty of evidence for the primary outcomes, as summarized in the Summary of Findings table, supports the effectiveness of the interventions. However, the Evidence Profile table highlights that several included studies were assessed as having a high risk of bias at the study level, mainly due to limitations in blinding and allocation procedures. While the effect estimates are generally consistent across studies, these methodological concerns indicate that the results should be interpreted with caution. Both the magnitude of the observed effects and the underlying certainty of evidence should be considered when applying these findings to clinical practice or further research.

Reorientation by family voice interventions reduced delirium incidence and duration. Munro et al. explained the effect firstly by enhanced feelings of security and comfort, reducing stress of critical care as a major contributor to delirium, and secondarily by frequent orientation, lowering disorientation as a symptom of delirium [24]. Also, Li et al.'s meta‐analysis about several family‐led interventions reported this hypothesis [27]. Kasapoglu et al. explained frequent reorientation by communicating information about the environment and daily news, increased attention due to listening to family voices and reorientation as being part of a prevention bundle including sleep hygiene and other factors [49]. Ma et al., Lin et al. as well as Liang et al. explained the preventive effect by sensory stimulation [46, 47, 48]. Another hypothesis is that reorientation by trustful voices can normalize the imbalance of neurotransmitters in delirium, especially acetylcholine, dopamine and likely serotonin, leading to calmness, orientation and emotional modulation [50]. Future research should evaluate this hypothesis.

Despite promising results supporting family integration, the optimal timing, frequency and duration of family voice interventions to prevent delirium remain unclear in this meta‐analysis.

The studies show significant variation in approach, frequency and dosage, with interventions delivered either through structured reorientation or unstructured conversations and via live visits [47] or pre‐recorded audios [24, 46, 49]. Furthermore, the study designs range from two‐arm [47] to three‐arm [24, 46, 49] experimental setups, which complicates comparability. Although this methodological diversity did not result in statistically significant heterogeneity, it represents a relevant limitation in interpreting the results.

Confounding factors in meta‐analysis, such as variations in patient populations, intervention effects, settings and statistical power, can greatly impact heterogeneity and the validity of combined results. Further research is needed to determine the optimal dosage and content. Positively, all studies employed similar measurement tools for delirium, enhancing results validity.

Guidelines in family‐centred care emphasize the role that relatives can play in the recovery process [51]. Open visiting policies to promote patients' emotional stability and orientation are recommended. Using family voices can aid reorientation and reduce delirium risk by proper involvement [51]. In addition, retrospective cohort data suggest that physical family presence and even telephone contacts are associated with shorter delirium duration, though effects on incidence may depend on patient population and admission characteristics [52]. Preventing delirium may also benefit families, but more research is needed to find the most effective type of reorientation by families [28].

Even though family voices have the potential to convey familiarity, orientation and emotional security, family voices can also trigger ambivalent or distressing associations. However, given the high rate of violence and abuse in relationships, the feasibility and safety of this intervention are questionable [53]. In complex and dynamic family relationships, such as divorce, conflict or problematic conditions, there is a risk that relatives' voices may not be perceived as reassuring but rather as a source of stress [54, 55]. In such cases, attempts at reorientation may be counterproductive. In the included studies, the authors of included RCT did not report such adverse events, and we cannot estimate a treatment effect. Other studies have addressed these psychosocial factors; questions have been raised about how to select the ‘right voices’, and whether some family constellations might be contraindicated. Despite these concerns, there is no evidence that family involvement harms ICU patients [13].

Another important consideration about the use of family voices is empathy. In the context of humanized delirium care, whether delivered by relatives or the professional team, empathic communication plays a central role [21]. Empathy can be considered an important part of the intervention in live reorientation. Contrary to this, empathy might be less relevant in pre‐recorded messages. This raises the question of how empathic reorientation messages are and how empathy can be conveyed within the intervention. Not all relatives have the emotional capacity for reassurance in the challenging environment of the ICU [56, 57]. Some may be overwhelmed themselves, whereas others may have only a limited understanding of the patient's situation [58]. This uncertainty raises further questions: How much empathy is necessary to achieve an effect, and at what point does it have a therapeutic impact? Future research should analyse the impact of empathy and reorientating messages.

It should also be noted that the systematic literature search revealed suitable study protocols and ongoing clinical studies, suggesting that an update to the review would be advisable in the future. Furthermore, social factors such as family closeness, relationship conflict, and the cultural significance of voice and communication need to be considered more thoroughly. Important steps for integration into nursing practice would include the development of standardized training concepts for relatives, clear selection criteria for the speakers involved, and ethical standards for dealing with stressful family systems. Future studies should also address how this intervention can be integrated into existing work processes.

### 4.1 Limitations

The meta‐analysis has several strengths and limitations. Strengths are the very strict inclusion criteria, evaluation of quality and certainty and low heterogeneity of included RCTs. Limitations are the small number of studies and the high risk of bias. Therefore, the results of the meta‐analysis must be viewed with caution. Regarding the available data, only meta‐analyses on the outcome of delirium could be considered, without sub‐analyses for the family voices, or any source, populations, different dosages and other factors sufficient to prevent delirium.

### 4.2 Practical Implication of the Intervention

It is recommended that practical implementation take place, based on the results of the analysis, while considering the patient's individual response and needs. This can be achieved by taking simple, barrier‐free measures to involve relatives in the care process. For example, flexible visiting hours could be introduced, and information material about delirium could be provided in waiting rooms.

## 5 Conclusions

Family voices might prevent and reduce the duration of delirium in critically ill patients. The involvement of family members in patient care not only promotes the patients' recovery but may also improve the psychological well‐being of the relatives. However, the heterogeneity in the intervention design and the potential for negative associations with family voices underscore the need for further research to optimize the use of this intervention. Standardized training and clear selection criteria for family involvement are essential for successful integration into clinical practice. Future studies should focus on addressing these gaps to enhance the effectiveness and applicability of family‐centred delirium care.

## Funding

The authors have nothing to report.

## Ethics Statement

Since this is a systematic review, an ethic approval is not necessary for this work. Registration: This search was pre‐registered in PROSPERO No.: CRD420251046497.

## Conflicts of Interest

The authors declare no conflicts of interest.

## Supporting information


**Supporting Information: S1.** Search strategy.
**Supporting Information: S2** PRISMA 2020 checklist.
**Supporting Information: S3** Detailed description of the study intervention.
**Supporting Information: S4** Additional meta‐analysis—delirium prevalence single intervention studies.
**Supporting Information: S5** Additional meta‐analysis—outcome delirium free days.
**Supporting Information: S6** Additional meta‐analysis—outcome delirium severity.
**Supporting Information: S7** Additional meta‐analysis—outcome days on mechanical ventilation.
**Supporting Information: S8** Additional meta‐analysis—outcome length of stay in the ICU.

## Data Availability

All the data are included in the manuscript or can be found in the Supporting Information.
