# Supplementary material for: The Effect of Family Voice Interventions on Delirium Incidence and Duration in Adult ICU Patients: A Systematic Review and Meta‐Analysis
Source: Nurs Crit Care. 2026 Apr 14;31(3):e70492. doi: 10.1111/nicc.70492 (PMC13080060; doi:10.1111/nicc.70492)

**SUPPLEMENT**

The Effect of Family Voice Interventions on Delirium Incidence and Duration in Adult ICU Patients: A Systematic Review and Meta-Analysis

**Content**

1. Search Strategies
2. PRISMA Checklist
3. Detailed description of the study Intervention
4. – 8. Additional Metaanalysis

**Supplement 1: Search Strategy (May 7^th^, 2025)**

**PUBMED (Filter: RCT, systematic reviews)**

("Intensive Care Units" OR "critical care" OR "intensive care unit" OR ICU OR “critical illness”)

AND

("Delirium" OR "acute confusion*" OR "acute brain dysfunction*" OR “encephalopathy”)

AND

("acustic Stimulation*" OR "Voice*" OR "sound therapy" OR "auditory input*" OR "auditory stimulation*" OR “speaking” OR “speech*” OR “voice reorientation” OR “vocal” OR “oral communication” OR “verbal” OR “delirium intervention*” OR “family-led intervention*” OR “family centered care”)

AND

("Family" OR “relatives” OR “loved-one*” OR “spouse*” OR “partner*” OR “next-of-kin” OR “husband*” OR “wife*” OR “child*” OR “Caregiver*” OR “parent*”)

**CINAHL (Filter: Randomized Controlled Trial, systematic review)**

(MH "Intensive Care Units" OR "critical care" OR "intensive care" OR ICU)

AND

(MH "Delirium" OR delirium OR "acute confusion" OR "brain dysfunction")

AND

((MH "Auditory Stimulation" OR voice recording OR "sound stimulation")

AND (family OR partner OR parent OR spouse OR children OR Caregiver OR husband OR wife OR relatives OR child OR loved-one)

**EMBASE**

("Intensive Care Units" OR "critical care" OR "intensive care unit" OR ICU OR “critical illness”)

AND

("Delirium" OR "acute confusion*" OR "acute brain dysfunction*" OR “encephalopathy”)

AND

("acustic Stimulation*" OR "Voice*" OR "sound therapy" OR "auditory input*" OR "auditory stimulation*" OR “speaking” OR “speech*” OR “voice reorientation” OR “vocal” OR “oral communication” OR “verbal” OR “delirium intervention*” OR “family-led intervention*” OR “family centered care”)

AND

("Family" OR “relatives” OR “loved-one*” OR “spouse*” OR “partner*” OR “next-of-kin” OR “husband*” OR “wife*” OR “child*” OR “Caregiver*” OR “parent*”)

**Cochrane Library**

(“intensive care” OR ICU OR "critical care")

AND

(delirium OR "acute confusion" OR "brain dysfunction")

AND

(family OR parent OR spouse OR children OR Caregiver)

AND

(voice OR voices OR recording OR "auditory stimulation")

**APA PsycNet (Filter: empirical study, systematic reviews)**

("intensive care" OR ICU OR "critical care")

AND

(delirium OR "acute confusion" OR "brain dysfunction")

AND

("auditory stimulation" OR voice OR voices OR "live voice" OR recording)

AND

(family OR parent OR spouse OR children OR Caregiver)

**Google Scholar**

Searching with relevant keywords like, delirium, family, audio, prevention

**Table Supplement 2: PRISMA Checklist**

| **Section and Topic** | **Item #** | **Checklist item** | **Location where item is reported** |
| --- | --- | --- | --- |
| **TITLE** | | |  |
| Title | 1 | Identify the report as a systematic review. | p. 1 |
| **ABSTRACT** | | |  |
| Abstract | 2 | See the PRISMA 2020 for Abstracts checklist. | p. 3 |
| **INTRODUCTION** | | |  |
| Rationale | 3 | Describe the rationale for the review in the context of existing knowledge. | p. 5 |
| Objectives | 4 | Provide an explicit statement of the objective(s) or question(s) the review addresses. | p. 5 |
| **METHODS** | | |  |
| Eligibility criteria | 5 | Specify the inclusion and exclusion criteria for the review and how studies were grouped for the syntheses. | p. 6, 7 |
| Information sources | 6 | Specify all databases, registers, websites, organisations, reference lists and other sources searched or consulted to identify studies. Specify the date when each source was last searched or consulted. | p. 6 |
| Search strategy | 7 | Present the full search strategies for all databases, registers and websites, including any filters and limits used. | Supp.1 |
| Selection process | 8 | Specify the methods used to decide whether a study met the inclusion criteria of the review, including how many reviewers screened each record and each report retrieved, whether they worked independently, and if applicable, details of automation tools used in the process. | p. 7,8 |
| Data collection process | 9 | Specify the methods used to collect data from reports, including how many reviewers collected data from each report, whether they worked independently, any processes for obtaining or confirming data from study investigators, and if applicable, details of automation tools used in the process. | p. 7 |
| Data items | 10a | List and define all outcomes for which data were sought. Specify whether all results that were compatible with each outcome domain in each study were sought (e.g. for all measures, time points, analyses), and if not, the methods used to decide which results to collect. | p.7 |
|  | 10b | List and define all other variables for which data were sought (e.g. participant and intervention characteristics, funding sources). Describe any assumptions made about any missing or unclear information. | p.7 |
| Study risk of bias assessment | 11 | Specify the methods used to assess risk of bias in the included studies, including details of the tool(s) used, how many reviewers assessed each study and whether they worked independently, and if applicable, details of automation tools used in the process. | p. 7 |
| Effect measures | 12 | Specify for each outcome the effect measure(s) (e.g. risk ratio, mean difference) used in the synthesis or presentation of results. | p. 7 |
| Synthesis methods | 13a | Describe the processes used to decide which studies were eligible for each synthesis (e.g. tabulating the study intervention characteristics and comparing against the planned groups for each synthesis (item #5)). | p. 7 |
|  | 13b | Describe any methods required to prepare the data for presentation or synthesis, such as handling of missing summary statistics, or data conversions. | n.e. |
|  | 13c | Describe any methods used to tabulate or visually display results of individual studies and syntheses. | p.7 |
|  | 13d | Describe any methods used to synthesize results and provide a rationale for the choice(s). If meta-analysis was performed, describe the model(s), method(s) to identify the presence and extent of statistical heterogeneity, and software package(s) used. | p. 7 |
|  | 13e | Describe any methods used to explore possible causes of heterogeneity among study results (e.g. subgroup analysis, meta-regression). | p.7, 8 |
|  | 13f | Describe any sensitivity analyses conducted to assess robustness of the synthesized results. | p. 7 |
| Reporting bias assessment | 14 | Describe any methods used to assess risk of bias due to missing results in a synthesis (arising from reporting biases). | P. 7 |
| Certainty assessment | 15 | Describe any methods used to assess certainty (or confidence) in the body of evidence for an outcome. | p. 7 |
| **RESULTS** | | |  |
| Study selection | 16a | Describe the results of the search and selection process, from the number of records identified in the search to the number of studies included in the review, ideally using a flow diagram. | p. 8 |
|  | 16b | Cite studies that might appear to meet the inclusion criteria, but which were excluded, and explain why they were excluded. |  |
| Study characteristics | 17 | Cite each included study and present its characteristics. | p.8, tab. 1 |
| Risk of bias in studies | 18 | Present assessments of risk of bias for each included study. | p. 9, tab. 2 |
| Results of individual studies | 19 | For all outcomes, present, for each study: (a) summary statistics for each group (where appropriate) and (b) an effect estimate and its precision (e.g. confidence/credible interval), ideally using structured tables or plots. | p. 8 |
| Results of syntheses | 20a | For each synthesis, briefly summarise the characteristics and risk of bias among contributing studies. | p. 9 |
|  | 20b | Present results of all statistical syntheses conducted. If meta-analysis was done, present for each the summary estimate and its precision (e.g. confidence/credible interval) and measures of statistical heterogeneity. If comparing groups, describe the direction of the effect. | p. 9, 10 |
|  | 20c | Present results of all investigations of possible causes of heterogeneity among study results. | p. 9,10 |
|  | 20d | Present results of all sensitivity analyses conducted to assess the robustness of the synthesized results. | p. 10 |
| Reporting biases | 21 | Present assessments of risk of bias due to missing results (arising from reporting biases) for each synthesis assessed. |  |
| Certainty of evidence | 22 | Present assessments of certainty (or confidence) in the body of evidence for each outcome assessed. | p. 9 |
| **DISCUSSION** | | |  |
| Discussion | 23a | Provide a general interpretation of the results in the context of other evidence. | p. 10,11 |
|  | 23b | Discuss any limitations of the evidence included in the review. | p. 12 |
|  | 23c | Discuss any limitations of the review processes used. | p. 12 |
|  | 23d | Discuss implications of the results for practice, policy, and future research. | p. 12 |
| **OTHER INFORMATION** | | |  |
| Registration and protocol | 24a | Provide registration information for the review, including register name and registration number, or state that the review was not registered. | p. 2 |
|  | 24b | Indicate where the review protocol can be accessed, or state that a protocol was not prepared. | p. 6 |
|  | 24c | Describe and explain any amendments to information provided at registration or in the protocol. | n. e.. |
| Support | 25 | Describe sources of financial or non-financial support for the review, and the role of the funders or sponsors in the review. | p. 2 |
| Competing interests | 26 | Declare any competing interests of review authors. | p. 2 |
| Availability of data, code and other materials | 27 | Report which of the following are publicly available and where they can be found: template data collection forms; data extracted from included studies; data used for all analyses; analytic code; any other materials used in the review. |  |

Abbreviations: *n.e.: not eligible*

**Table Supplement 3: Detailed description of the interventions**

| **Study** | **ICU Type** | **Patient on MV n(%)^a^** | **Definition of Family** | **Detailed Description of the Voice** |
| --- | --- | --- | --- | --- |
| Kasapoğlu & Enҫ, 2022 | General  Other: pulmonary | 8 (7,5) | voice of a family member (not precisely defined) | Group 2:Voice recorded; (orientation message); Voice present (newspaper) |
| Lin et al., 2023 | surgical cardiovascular | n.r. | primary  responsibility for caring for  the patient, such as spouses, children, brothers, or sisters, and other family members | speaker present at ICU Psychological support:  emotional support, worshipping the  patients feelings, feelings of security,  positive attitude to the disease Orientation: Who, Where, When, What, Why and how Cognitive simulation: talking about family life, showing photographs, personalizing the bedside Participation in ICU care; (prevention sensory prevention: using visual and auditory devices) |
| Ma et al., 2025 | other | n.r. | (1) adults (older than or  equal to 18years); (2)lived  with the patient; and(3)was  the primary caregiver among the patient’s family. | Group A structured family auditory stimulation: using the familys` name to the patient, introducing the speaker and his relation to the patient, time orientation (date/time, days of ICU stay), current location, reason and process of ICU stay, explanation of special environment, equipment and treatment, patients current condition, status of surgery, expressing love, concern, security for the patient, persuading to trust in hospital staff and to cooperate, relieving psychological burden (worries about other persons or costs), telling about daily life, recalling friends and co-workers, specific encouragment: condition stabilisation, plans and wishes for time after discharge, talking about patients interests and concerns |
| Munro et al., 2017 | other | 2,1 (+ 5,5) days | a family member of the  family’s choice | Group 2: familiar voices recorded, order of the sentences randomly changed every hour - addressing patient by name, speaker  introduction and explaining the recording 1. Do not be scared.(It is OK; You are a patient at Tampa General Hospital; Your nurses and doctors are here looking after you; It is loud and noisy because of the machines helping you get better; You have some wires and tubes in place to help you recover; You may have something on your wrists to keep you from pulling at the wires and tubes by accident; You can’t talk right now because of your breathing tube, but the nurses know you might be uncomfortable and are giving you medicine for that; Please try to be calm and patient as the nurses and doctors work to get you feeling better; All of our family know you’re here and we are in and out, looking after you too) |
| Sprüngli et al., 2025 | Neurological ICU | 5.3 (3.3-8.0) | Has a close personal relationship to the patient; Family member was chosen based on availability, willingness to participate | Audio recordings of patients` relatives (1-minute script with breathing commands) were edited into 10-minute files with 15-secnd breaks, played three times daily (morning, midday, evening) via headphones during assisted ventilation. The sham control used muted recordings. |
| Munro et al., 2025 | Medical, surgical, trauma, Neurologic, cardiovascular | n.r. | Is willing and able to record scripted messages, speaks English or Spanish, Is chosen by patient or their legal representative to record the messages | Standardized 2-minte voice messages recorded by a family member (English/Spanish); Reorientation to the ICU environment, mention of healthcare staff/family, and temporal cues; messages were played hourly from 9AM to 4 M for up to 5 days (or until ICU discharge); up to 8 doses/day; the messages were applied through wireless speaker placed near the patient, automatic playback |
| Liang et al., 2023 | surgical | 110 (72.4) | Individuals capable of offering emotional and orienting support to the patient | Daily 45-minute sensory stimulation (auditory: family-recorded reorientation messages + light music; visual: family photos, written messages, reading materials) for 7 days, tailored to patient`s condition (unconscious/auditory-only; intubated/visual + auditory; extubated/interactive). Family prepared materials remotely due to COVID-19 restrictions |

Abbreviations: ICU Intensive Care Unit, MV mechanically ventilation, n.r. not reported

^a^ reported as mean (standard deviation), or median (interquartil range)

Figure Supplement 4: Delirium Prevalence Single Intervention studies*


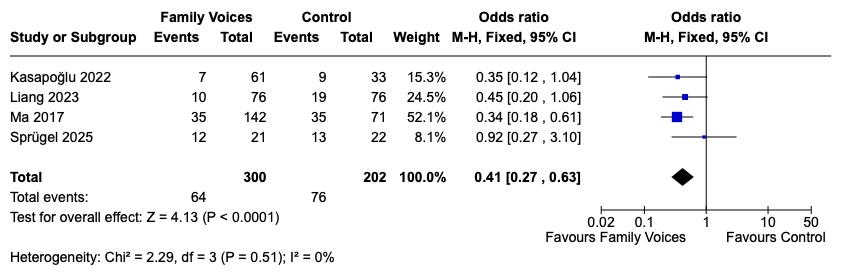


**Family Voices always includes both intervention groups, if there were more than one in the study*

Figure Supplement 5: Delirium free days


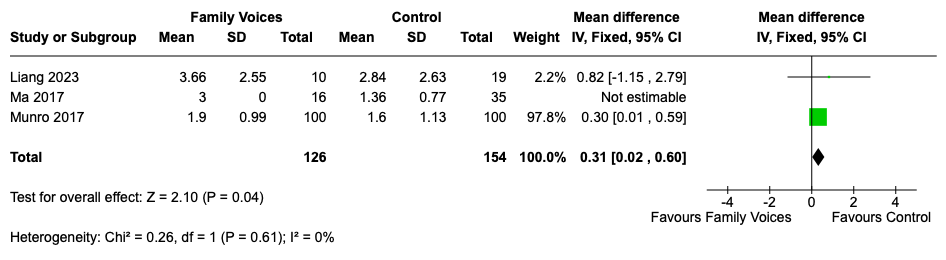


Figure Supplement 6: Delirium Serverity


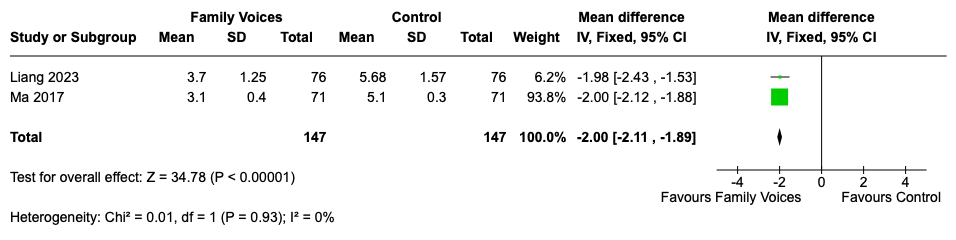


Figure Supplement 7: Days on Mechanical Ventilation


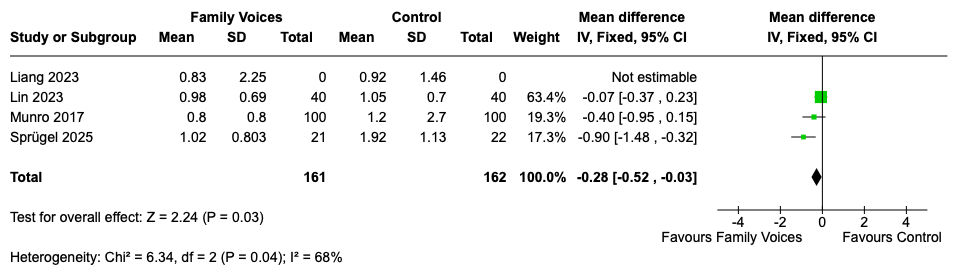


Figure Supplement 8: Length of stay in the ICU


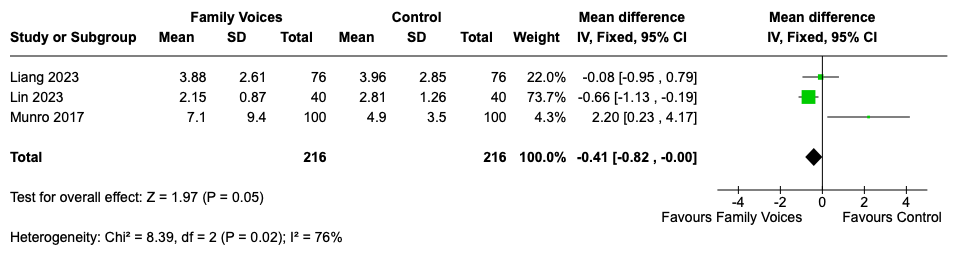

Supplement: Supplementary file 1 — Supporting Information: S1. Search strategy. Supporting Information: S2 PRISMA 2020 checklist. Supporting Information: S3 Detailed description of the study intervention. Supporting Information: S4 Additional meta‐analysis—delirium prevalence single intervention studies. Supporting Information: S5 Additional meta‐analysis—outcome delirium free days. Supporting Information: S6 Additional meta‐analysis—outcome delirium severity. Supporting Information: S7 Additional meta‐analysis—outcome days on mechanical ventilation. Supporting Information: S8 Additional meta‐analysis—outcome length of stay in the ICU. [file NICC-31-0-s001.docx]
